# Roads to ruin: conservation threats to a sentinel species across an urban gradient

**DOI:** 10.1002/eap.1615

**Published:** 2017-10-18

**Authors:** Blake E. Feist, Eric R. Buhle, David H. Baldwin, Julann A. Spromberg, Steven E. Damm, Jay W. Davis, Nathaniel L. Scholz

**Affiliations:** ^1^ Conservation Biology Division Northwest Fisheries Science Center National Marine Fisheries Service, NOAA 2725 Montlake Boulevard East Seattle Washington 98112 USA; ^2^ Quantitative Consultants, Inc. Under contract to Northwest Fisheries Science Center National Marine Fisheries Service, NOAA 2725 Montlake Boulevard East Seattle Washington 98112 USA; ^3^ Environmental and Fisheries Sciences Division Northwest Fisheries Science Center National Marine Fisheries Service, NOAA 2725 Montlake Boulevard East Seattle Washington 98112 USA; ^4^ Washington Fish and Wildlife Office United States Fish and Wildlife Service 510 Desmond Drive SE Lacey Washington 98392 USA

**Keywords:** Bayesian, ecotoxicology, Pacific salmon, restoration, Stan, stormwater, structural equation modeling, urbanization

## Abstract

Urbanization poses a global challenge to species conservation. This is primarily understood in terms of physical habitat loss, as agricultural and forested lands are replaced with urban infrastructure. However, aquatic habitats are also chemically degraded by urban development, often in the form of toxic stormwater runoff. Here we assess threats of urbanization to coho salmon throughout developed areas of the Puget Sound Basin in Washington, USA. Puget Sound coho are a sentinel species for freshwater communities and also a species of concern under the U.S. Endangered Species Act. Previous studies have demonstrated that stormwater runoff is unusually lethal to adult coho that return to spawn each year in urban watersheds. To further explore the relationship between land use and recurrent coho die‐offs, we measured mortality rates in field surveys of 51 spawning sites across an urban gradient. We then used spatial analyses to measure landscape attributes (land use and land cover, human population density, roadways, traffic intensity, etc.) and climatic variables (annual summer and fall precipitation) associated with each site. Structural equation modeling revealed a latent urbanization gradient that was associated with road density and traffic intensity, among other variables, and positively related to coho mortality. Across years within sites, mortality increased with summer and fall precipitation, but the effect of rainfall was strongest in the least developed areas and was essentially neutral in the most urbanized streams. We used the best‐supported structural equation model to generate a predictive mortality risk map for the entire Puget Sound Basin. This map indicates an ongoing and widespread loss of spawners across much of the Puget Sound population segment, particularly within the major regional north‐south corridor for transportation and development. Our findings identify current and future urbanization‐related threats to wild coho, and show where green infrastructure and similar clean water strategies could prove most useful for promoting species conservation and recovery.

## Introduction

River networks in landscapes throughout the world are under increasing pressures from urban and suburban development. The global human population has more than doubled over the past 50 yr, intensifying upward and outward growth in urban areas (Seto et al. 2012, Frolking et al. 2013, Barragán and de Andrés 2015). Increasing imperviousness is a consistent but complex driver for biological decline in aquatic habitats. This is classically termed the urban stream syndrome, wherein physical, biological, and chemical forms of habitat degradation collectively reduce the diversity and abundance of aquatic species (Paul and Meyer 2001, Walsh et al. 2005, Bernhardt and Palmer 2007, Grimm et al. 2008, Schueler et al. 2009, Pickett et al. 2011, Canessa and Parris 2013). The relative role of degraded water quality in the syndrome is poorly understood, in part because hundreds or even thousands of distinct chemical contaminants in urban stormwater runoff have never been toxicologically characterized.

In northwestern North America, Pacific salmon (*Oncorhynchus* spp.) are iconic in terms of their cultural, economic, and ecological significance. They are central to the identity and traditional practices of indigenous peoples, vital for recreational and commercial fisheries, and keystone species (Willson and Halupka 1995, Kaeriyama et al. 2012, LeRoy et al. 2015) for inland ecosystems as sources of marine‐derived nutrients (Naiman et al. 2002, Helfield and Naiman 2006). Coho salmon (*O. kisutch*) in particular are a prominent sentinel species for the urban stream syndrome (Scholz et al. 2011) and the impetus for emerging green infrastructure methods for filtering pollutants to improve water quality in salmon spawning and rearing habitats (McIntyre et al. 2015, Spromberg et al. 2016). Adult coho return from the ocean to spawn in basins that span many of the largest metropolitan areas in the Pacific Northwest and Canada (e.g., Portland, Oregon; Seattle, Washington; Vancouver, British Columbia). Juveniles typically spend ~1.5 yr in freshwater before migrating seaward to the estuary. During freshwater residency the adults, embryos, and juveniles depend upon the small, first‐ through third‐order streams that are the most vulnerable to land use change (Allan 2004).

Adult coho salmon are exceptionally sensitive to the harmful effects of toxic urban runoff. Field surveys spanning more than a decade have shown very high rates of mortality in urban streams from the central Puget Sound Basin (Scholz et al. 2011). Affected adult males and gravid females become disoriented and show surface swimming, gaping, a loss of equilibrium, and finally death on a timescale of a few hours. Extensive forensic research has ruled out stream temperature, dissolved oxygen, spawner condition, tissue pathology, pathogens or disease, and other factors commonly associated with fish kills in freshwater habitats (Scholz et al. 2011), which suggests that toxicants found in stormwater runoff are the most likely culprit. Consistent with this, direct exposures to untreated urban stormwater reproduce the mortality syndrome in adult coho, and this toxicity is prevented by pre‐treatment with bioinfiltration to remove chemical contaminants (Spromberg et al. 2016). Loss rates to die‐offs are typically high, e.g., 60–90% of an entire fall run within a given urban stream. Initial modeling has shown that wild Puget Sound coho, presently a species of concern under the U.S. Endangered Species Act, cannot maintain population abundances at such high mortality rates (Spromberg and Scholz 2011). Mortality corresponds with urbanization within a basin, and many restoration projects in lowland streams have the potential to become ecological traps (Feist et al. 2011). These threats to the Puget Sound coho evolutionarily significant unit (ESU; defined as a group of populations that (1) are substantially reproductively isolated from conspecific populations and (2) collectively represent an important component in the evolutionary legacy of the species [Waples 1991]) can be expected to increase in the years ahead with expanding regional human population growth and development.

To date, our understanding of the association between coho die‐offs and imperviousness at the landscape scale was derived from a spatial analysis of a few (*n *=* *6) highly urbanized streams and one non‐urban stream (Feist et al. 2011). The previous analysis was geographically restricted, did not consider possible interactions between landscape and climate, and did not include a full probabilistic accounting of uncertainty in predictions (Clark 2005). Here we present an expanded analysis based on field surveys of 51 distinct coho salmon spawning reaches across a gradient of urbanization in the Puget Sound basin.

Lack of independence among potential predictor variables (i.e., multicollinearity) is a characteristic of many ecological data sets and presents challenges for modeling and causal inference (Dormann et al. 2013). For example, landscape attributes such as impervious surfaces, road and traffic density, and human population are expected to covary at relevant scales because they are all indicators of the underlying process of urban development. Given this challenge, one approach is to use model selection (Burnham and Anderson 2002) or shrinkage priors (Carvalho et al. 2012, Hooten and Hobbs 2015) to identify a “sparse” model that includes only relevant predictors, with the coefficients of other nuisance variables either fixed at zero or shrunk to small values to avoid overfitting the data. We do not pursue these approaches here because the structural, and often severe, correlations among the landscape variables of interest make it dubious *a priori* that such data can reveal a distinct and specific cause for coho die‐offs.

Instead, we seek to reduce the high‐dimensional, unwieldy predictor variable space by taking advantage of the partially redundant signal in the raw landscape attributes to construct one or more composite indicators that are calibrated to predict mortality risk. A common approach to this problem is to use principal component analysis (PCA) or other ordination procedures to combine the correlated predictor variables into orthogonal axes, and then to use the dominant PCs as inputs in a regression model (Dormann et al. 2013). The axes found by such *unsupervised dimension reduction* techniques may be optimal in some sense for uncovering the structure of the predictor space, but they are not tuned to optimally predict the response variable of interest (Jolliffe 1982). By contrast, *supervised dimension reduction* simultaneously finds the hidden structure among a suite of predictor variables and models the relationship between the derived trends or gradients and the response (Yu et al. 2006, Gönen 2013). Structural equation modeling (SEM; Grace et al. 2010) is a natural framework for supervised dimension reduction, as it allows the specification of flexible, general causal network models that can include latent variables representing underlying factors that correspond to multiple observed indicators. Embedding SEM within a Bayesian inference paradigm (Lee and Song 2012) permits formal accounting of various sources of stochasticity, including hierarchical structure (e.g., observations grouped by site).

Here we use Bayesian SEM to distill a high‐dimensional suite of correlated landscape attributes into a single latent indicator that is designed to predict the observed rates of coho spawner mortality, in conjunction with seasonal rainfall. Our goals were twofold: (1) refine our understanding of which types of human activities are most closely associated with the recurrent mortality phenomenon and (2) identify areas where urban stormwater mitigation is most needed to benefit ongoing coho conservation efforts. Our approach efficiently identified underlying signals of land‐use and rainfall effects on salmon mortality, given noisy observations of multiple imperfect and partially redundant proxy variables. The improved, spatially explicit forecasts of coho mortality hotspots incorporate rigorous, straightforward propagation of uncertainty. Our findings are discussed in the context of motor vehicles as a primary driver for coho die‐offs.

## Materials and Methods

### Field surveys for premature coho spawner mortality

**Figure 1:**
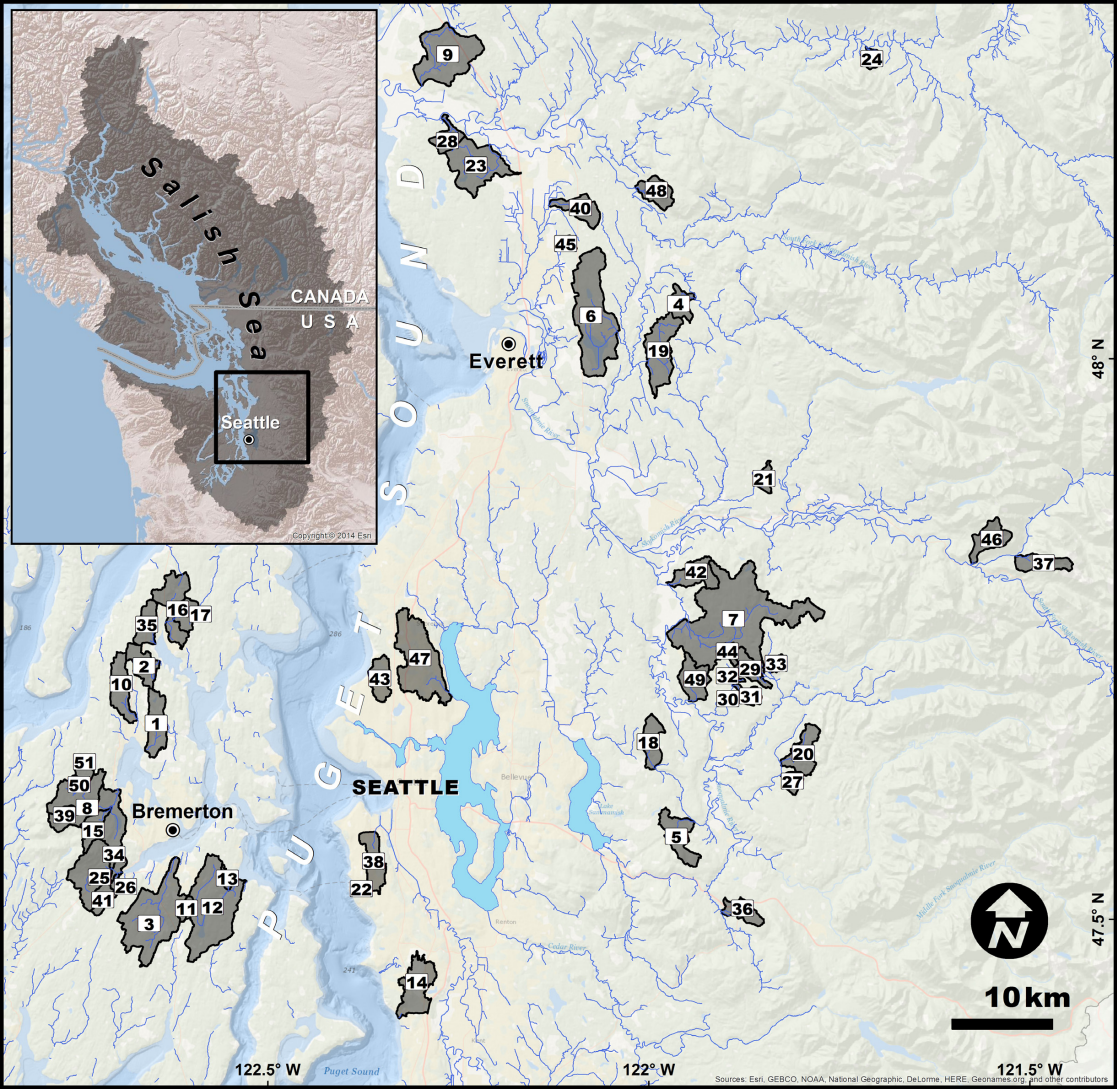
Study region and location of site subbasins within the Salish Sea basin (inset map gray region). (1) Barker, (2) Big Scandia, (3) Blackjack, (4) Bosworth, (5) Canyon, (6) Catherine, (7) Cherry, (8) Chico, (9) Church, (10) Clear WF, (11) Cool, (12) Curley, (13) Curley Tributary, (14) Des Moines, (15) Dickerson, (16) Dogfish, (17) Dogfish NF, (18) Dry, (19) Dubuque, (20) E.F. Griffin, (21) Eager Beaver, (22) Fauntleroy, (23) Fish, (24) Fortson, (25) Gorst, (26) Gorst Tributary, (27) Grizzly, (28) Happy Hollow, (29) Harris, (30) Harris Tributary B, (31) Harris Tributary C, (32) Harris Tributary D, (33) Index, (34) Jarstad, (35) Johnson, (36) Lake, (37) Lewis, (38) Longfellow, (39) Lost, (40) MF Quilceda, (41) Parish, (42) People's, (43) Pipers, (44) Pond, (45) Ross, (46) Son of Deer, (47) Thornton, (48) Valhalla, (49) Weiss, (50) Wildcat, (51) Wildcat Tributary. [Color figure can be viewed at wileyonlinelibrary.com]

Overall rates of mortality across a fall spawning season (generally October–December) were enumerated as the proportion of egg‐retaining female carcasses identified during field surveys of freshwater spawning habitats (Scholz et al. 2011). Males and females are equally affected by the mortality phenomenon, but the pre‐spawning condition is more reliably diagnosed in the latter. Carcasses with evident signs of predation (e.g., from river otters) were not included in mortality counts. For the present study, trained fisheries biologists from NOAA Fisheries, the U.S. Fish and Wildlife Service, the Wild Fish Conservancy, and the Suquamish and Stillaguamish Tribes (Appendix S1: Table S1) surveyed 51 distinct spawning reaches using established protocols from 2000 to 2011 (Scholz et al. 2011). The spawning locations spanned a gradient of urbanization in Puget Sound (Fig. 1). Certain streams were surveyed for only a single spawning season, while others were surveyed across multiple years (Appendix S1: Table S1).

### Geospatial data layers

Geospatial data layers were obtained from the U.S. Census Bureau, the U.S. Homeland Security Infrastructure Program, NOAA's Coastal Change Analysis Program, the U.S. Geological Survey's National Land Cover Database, and other public sources (Appendix S1: Table S2). The layers included precipitation, land use and land cover, imperviousness, roadways, human population density, and the extent of NOAA‐documented physical habitat restoration within a given stream basin.

### Spatial analyses

We used Esri ArcGIS software suite (v. 10.1 Redlands, CA, USA), Esri ArcView (v. 3.2a), and QGIS (v. 2.84 Open Source Geospatial Foundation, Beaverton, OR, USA) for all spatial analyses. The upstream and downstream locations of each coho mortality survey site were georeferenced onsite using handheld GPS units, exported to ArcGIS, and overlaid on a fine‐grained stream network (NHDPlus 2012b). The network was then manually clipped to these upstream and downstream points to create a stream reach for each site. These reach segments were used to delineate the stream subbasin associated with each of the 51 study sites by combining and editing polygons from an existing geospatial data layer (NHDPlus 2012a), and by modifying these polygons as necessary using various digital elevation models (DEM) as a topography guide (PSLC 2010, USGS 2014). We then intersected the corresponding subbasin boundary for each of the 51 sites with each of the aforementioned geospatial data layers. We processed each of the data layers using a variety of approaches, which are described in detail in Appendix S1: Section S1.

### Multilevel structural equation model

**Figure 2:**
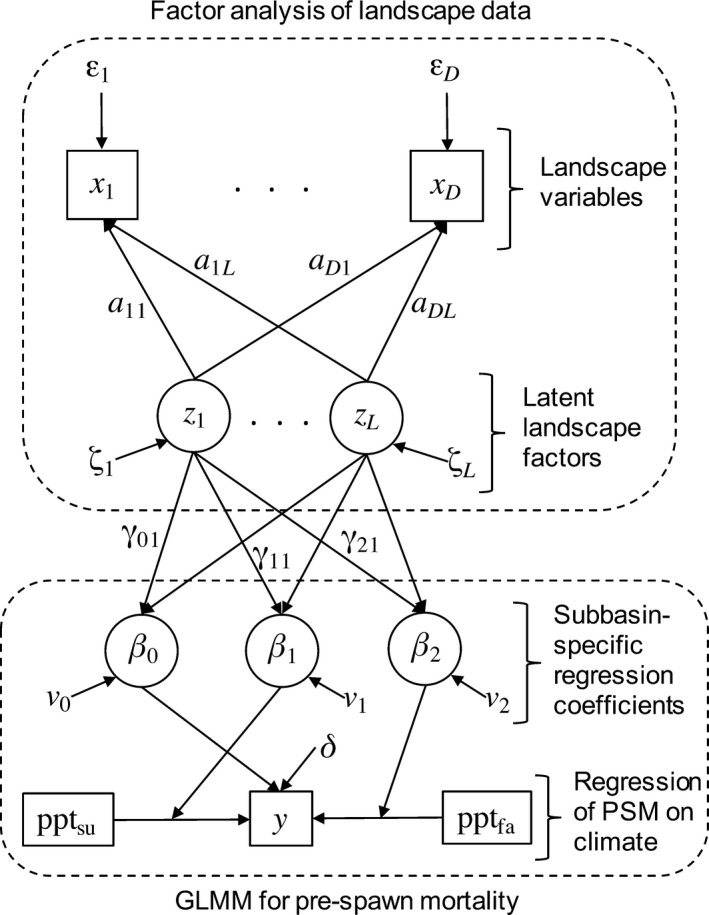
Structural equation model (SEM) linking land use/land cover and climate to coho salmon pre‐spawn mortality. The model can be interpreted as a factor analysis for landscape attributes coupled to a generalized linear mixed‐effects model (GLMM) for mortality. Observed variables are in rectangles, latent variables and random effects are in circles, and variables without shapes represent stochastic error terms. Arrows pointing from predictor to response variables represent functional relationships parameterized by coefficients shown beside each arrow. (Note that γ_0*L*_, γ_1*L*_, and γ_2*L*_ are omitted for clarity.) Arrows pointing from variables to other arrows indicate that the variable is a coefficient for a functional relationship. See *Factor analysis for landscape indicators and Generalized linear mixed‐effects model for pre‐spawn mortality* for variable and parameter definitions. In the final model, *L *=* *1 and we refer to *z*_1_ = *z* as a latent “urbanization gradient.”

We used structural equation modeling to relate landscape attributes and seasonal rainfall to observed rates of coho spawner mortality. A structural equation model (SEM) is a network model that represents hypothesized pathways linking observed and unobserved (latent) variables (Grace et al. 2010, 2012, Lee and Song 2012). Variables are linked by regression‐like relationships, and the data (observed variables) are used to estimate the parameters of these relationships along with the values of the latent variables. The structure of our SEM can be interpreted heuristically as a factor analysis for the landscape variables, coupled to a generalized linear mixed‐effects model (GLMM; Bolker et al. 2009) for spawner mortality (Fig. 2). The climatic variables (total summer and fall precipitation) were data‐level predictors of mortality risk, while the latent factors representing the underlying landscape conditions in each subbasin were predictors in the group‐level (i.e., subbasin‐level) model for the regression coefficients.

### Factor analysis for landscape indicators

The factor‐analytic component of the model projects the high‐dimensional space of *D* mutually correlated landscape variables (*x*_1_, …, *x*_*D*_) onto a lower‐dimensional subspace of *L* latent factors (*z*_1_, …, *z*_*L*_) that are mutually independent by construction. In the simplest case, each *x* is conditionally normally distributed, such that

(1) $$\begin{array}{cc} & x_{sj}=a_{0j}+\sum_{l=1}^{L}a_{jl}z_{sl}+\varepsilon_{sj}\\ & z_{sl}∼N\left(0,1\right)\\ & \varepsilon_{sj}∼N\left(0,\sigma_{j}\right), \end{array}$$

where *x*_*sj*_, the observed value of variable *j* in subbasin *s *∈ {1,…, *S*}, is represented as a regression on the latent factors with intercept *a*_0*j*_ and slopes (i.e., factor loadings) *a*_*jl*_ for *j *∈ { 1,…, *D*} and *l *∈ { 1,…, *L*}, and a residual error with standard deviation σ_*j*_. For this model to be identifiable, *L* must be less than *D*/2 (Geweke and Zhou 1996). Identification constraints are discussed further in Appendix S1: Section S2.

This framework can be extended to non‐normally distributed data using a formulation analogous to generalized linear models (GLMs; Yu et al. 2006). Specifically, a link function *g*(μ) is used to transform the mean μ, which is then described by a linear regression on the link scale

(2) $$\begin{array}{cc} & g\left(\mu_{sj}\right)=a_{0j}+\sum_{l=1}^{L}a_{jl}z_{sl}\\ & z_{sl}∼N\left(0,1\right)\\ & x_{sj}∼f_{j}\left(g^{-1}\left(\mu_{sj}\right),\phi_{j}\right), \end{array}$$

where the intercepts and factor loadings are as in Eq. (1), but the observation *x*_*sj*_ has an exponential‐family probability distribution with mean *g* ^−1^(μ_*sj*_) and a family‐specific dispersion parameter ϕ_*j*_.

Our analysis included a combination of normal and non‐normal landscape variables. For proportional composition data types (e.g., land cover classes and impervious surface cover), we used logistic normal distributions (Aitchison 2003) after bounding the values away from 0 or 1 by a small increment (10^−4^). All other landscape attributes are nonnegative continuous (or approximately continuous in the case of restoration site and human population density) variables with typically highly skewed distributions and many zero observations. We bounded these variables away from 0, scaled them to have unit variance, and then modeled them as in Eq. (2) using gamma distributions parameterized by the mean (via a log link function) and the shape ϕ_*j*_.

### Generalized linear mixed‐effects model for pre‐spawn mortality

The second component of the SEM (Fig. 2) is a regression model that relates the latent landscape factors and seasonal precipitation to the observed frequencies of coho mortality. This takes the familiar form of a GLMM or multilevel regression model with a binomial error distribution and logit link function (Bolker et al. 2009). The “full” data‐level model for an observation *y*_*is*_, the number of prematurely dead female coho out of *n*_*is*_ total female spawners sampled in subbasin *s* in year *i*, is

(3) $$\begin{array}{cc} & y_{is}∼\text{Bin}\left(n_{is},p_{is}\right)\\ & \text{logit}\left(p_{is}\right)=\beta_{0}^{\left(s\right)}+\beta_{1}^{\left(s\right)}{\text{ppt}}_{\mathrm{su},is}+\beta_{2}^{\left(s\right)}{\text{ppt}}_{\mathrm{fa},is}+\delta_{is} \end{array}$$

where the logit‐linear model for mortality risk *p*_*is*_ includes effects of summer and fall precipitation and a residual error term, δ_*is*_ ~ *N*(0,σ_δ_), that accounts for overdispersion relative to the binomial distribution. We found strong support for the overdispersion term (see *Model selection*), and therefore included it in subsequent stages of model development.

The second hierarchical level of the GLMM consists of linear models for one or more of the subbasin‐specific coefficients in Eq. (3). The most general formulation is a varying‐intercept, varying‐slopes model

(4) $$\begin{array}{cc} & \beta_{0}^{\left(s\right)}=\gamma_{00}+\sum_{l=1}^{L}\gamma_{0l}z_{sl}+v_{0s}\\ & \beta_{1}^{\left(s\right)}=\gamma_{10}+\sum_{l=1}^{L}\gamma_{1l}z_{sl}+v_{1s}\\ & \beta_{2}^{\left(s\right)}=\gamma_{20}+\sum_{l=1}^{L}\gamma_{2l}z_{sl}+v_{2s}, \end{array}$$

where the hyper‐parameters for each logistic regression coefficient β_*k*_ include an intercept γ_*k*0_, slopes γ_*kl*_ that define how the coefficient varies among subbasins as a function of each of the *L* latent landscape factors *z*_*l*_, and a hyper‐variance for the subbasin‐level random effect $\nu_{ks}∼N\left(0,\sigma_{\beta_{k}}\right)$. For simplicity, we assume the random effects *v*_0*s*_, *v*_1*s*_, and *v*_2*s*_ are uncorrelated. By including subbasin‐level effects, we account for potential nonindependence among repeated mortality observations at the same sites, thus avoiding pseudoreplication. This also allows for the possibility that different spawning habitats may have different relationships between seasonal rainfall and coho mortality, and that the underlying landscape conditions may explain some of these differences. From the data‐level perspective, the slopes γ_0*l*_ for the intercept β_0_ can be interpreted as the “main effects” of landscape factors on mortality risk, while the slopes γ_1*l*_ and γ_2*l*_ represent interactions between precipitation and landscape variables because they modify the precipitation coefficients β_1_ and β_2_, respectively.

### Parameter estimation

The SEM was fit to the landscape and coho mortality data using a Bayesian framework (Lee and Song 2012). We used vague or noninformative priors for most hyper‐parameters (see Appendix S1: Section S2 for details on the construction of the joint posterior distribution). Samples were drawn from the joint posterior distribution using Hamiltonian Monte Carlo (HMC) tuned by the no‐U‐turn sampler (NUTS; Hoffman and Gelman 2011) implemented in Stan version 2.12.0 (Carpenter et al. 2016) via the rstan package in R version 3.3.1 (R Development Core Team 2016). HMC/NUTS is a Markov chain Monte Carlo (MCMC) algorithm that efficiently generates proposals with low autocorrelation and is well suited for complex, high‐dimensional, posterior geometries.

### Model selection

We used model selection approaches to compare the strength of evidence for restricted models or special cases of the general SEM. In principle, this strategy can be used to identify the dimension *L* of the latent factor space most consistent with the data (Lopes and West 2004). We found, however, that models with *L *>* *1 had pathologies such as nontrivial posterior multimodality, strongly suggesting that higher‐dimensional factor spaces are inconsistent with the underlying correlation structure of the landscape variables (Erosheva and Curtis 2011). Our subsequent analyses were therefore restricted to single‐factor models, focusing on the effects of the latent factor *z* (henceforth “urbanization”) and seasonal rainfall on coho mortality risk in the GLMM‐like component of the model. Specifically, we evaluated 18 candidate models constructed by setting various combinations of the regression coefficients β_*k*_ in the data‐level model for coho mortality (Eq. (3)) and the coefficients γ_*kl*_ in the subbasin‐level model (Eq. (4)) equal to zero (Table 1). Each coefficient $\beta_{k}^{\left(s\right)}$ could be either (1) fixed at zero for all subbasins *s* (except for the intercept $\beta_{0}^{\left(s\right)}$, which was always estimated); (2) estimated with a hyper‐mean γ_*k*0_ and hyper‐SD $\sigma_{\beta_{k}}$ but without any effect of urbanization; or (3) estimated and allowed to vary in response to urbanization with slope γ_*k*1_. From the data‐level perspective, comparing (1) vs. (2) tests the effect of rainfall on mortality risk, while comparing (2) vs. (3) tests the effect of urbanization (in the case of $\beta_{0}^{\left(s\right)}$) or a rainfall by urbanization interaction (in the case of $\beta_{1}^{\left(s\right)}$ and $\beta_{2}^{\left(s\right)}$).

**Table 1:** Structural equation model selection based on two Bayesian information criteria (W the Watanabe‐Akaike information criterion [WAIC] and approximate leave‐one‐out cross‐validation score [PSIS‐LOO])

Intercept (β_0_)	Summer rain (β_1_)	Fall rain (β_2_)	$\overset{¯}{D}$	*p*_WAIC_	Δ_WAIC_	*p*_PSIS‐LOO_	Δ_PSIS‐LOO_
1	*z*	0	437.21	30.38	1.01 (4.88)	35.35	0
*z*	*z*	1	431.33	34.10	0	40.13	1.13 (5.89)
*z*	*z*	0	440.66	30.21	4.18 (2.98)	34.76	2.32 (4.50)
*z*	1	0	443.48	29.93	6.41 (4.18)	33.96	3.52 (6.12)
1	1	0	441.46	30.77	5.55 (5.08)	35.36	3.78 (3.72)
*z*	*z*	*z*	433.30	34.24	2.16 (2.25)	40.65	4.04 (6.06)
1	*z*	1	432.87	34.57	2.23 (4.49)	41.12	4.38 (2.64)
1	*z*	*z*	432.77	34.90	2.62 (4.53)	41.34	4.55 (2.79)
*z*	1	1	436.94	33.89	5.17 (3.67)	39.50	5.44 (6.59)
1	1	1	437.96	33.81	6.12 (5.75)	39.87	7.29 (5.01)
*z*	0	1	438.11	33.44	5.94 (6.73)	39.75	7.62 (9.68)
1	0	1	438.78	33.30	6.38 (7.08)	39.50	7.83 (7.34)
*z*	0	*z*	436.47	34.45	5.85 (6.86)	41.36	8.72 (10.07)
1	0	*z*	435.93	34.47	5.42 (6.41)	41.90	9.33 (7.80)
*z*	1	*z*	438.78	35.11	8.63 (4.30)	41.05	9.57 (7.19)
1	1	*z*	436.78	35.33	7.19 (5.26)	42.40	10.39 (5.41)
*z*	0	0	454.72	26.87	13.81 (8.42)	30.75	10.64 (10.83)
1	0	0	451.94	28.06	12.72 (8.41)	32.75	11.15 (9.22)

*Notes*

Candidate models for coho mortality differ in whether the subbasin‐specific intercept and slopes (seasonal rainfall effects) are fixed at zero (0), estimated as a mean only (1), or modeled as a function of urbanization (*z*). The latter two cases also include random subbasin effects. All models include an intercept. Posterior mean deviance ($\overset{¯}{D}$), complexity penalty (*p*), and score relative to the best model on each criterion (Δ, where smaller values indicate stronger support and the SE is in parentheses) are shown.

We used multiple, complementary model‐selection approaches (Hooten and Hobbs 2015). Information criteria were used to estimate out‐of‐sample predictive performance by penalizing the within‐sample fit for optimism (i.e., overfitting). We calculated the Watanabe‐Akaike information criterion (WAIC) and approximate leave‐one‐out cross‐validation score (PSIS‐LOO) for each of the 18 candidate models (Vehtari and Ojanen 2012, Vehtari et al. 2015). WAIC is a Bayesian generalization of the familiar Akaike information criterion, while PSIS‐LOO approximates the leave‐one‐out posterior predictive density (i.e., the probability of an observation, conditioned on all the other observations in the sample) by Pareto‐smoothed importance resampling (Vehtari et al. 2015). Because our focus is on predicting coho mortality risk, we calculated WAIC and PSIS‐LOO from just the posterior predictive distribution of the mortality frequency data (i.e., the last line in Eq. A5) after marginalizing out the data‐level random effects (the next‐to‐last line in Eq. A5) by Monte Carlo integration.

As a more realistic test of predictive performance, we used *K*‐fold cross‐validation (Hooten and Hobbs 2015, Vehtari et al. 2015) structured by years or by sites. Because cross‐validation is computationally intensive, we restricted the candidate set to four models representing minimal or maximal complexity and two intermediate structures supported by WAIC and PSIS‐LOO (Table 2). Details of the cross‐validation procedures are described in Appendix S1: Section S3.

**Table 2:** Structural equation model selection based on *K*‐fold cross‐validation over years or subbasins

Intercept (β_0_)	Summer rain (β_1_)	Fall rain (β_2_)	Leave years out		Leave subbasins out	
			Δ_ELPD_	SE	Δ_ELPD_	SE
*z*	*z*	*z*	6.72	8.75	0.00	0.00
*z*	*z*	1	4.48	8.63	8.31	2.68
1	*z*	0	0.00	0.00	45.61	12.90
1	0	0	16.36	9.58	62.22	13.61

*Notes*

Candidate models for coho mortality differ in whether the subbasin‐specific intercept and slopes (seasonal rainfall effects) are fixed at zero (0), estimated as a mean only (1), or modeled as a function of urbanization (*z*). The latter two cases also include random subbasin effects. All models include an intercept. Expected log predictive deviance (ELPD), relative to the best model (Δ_ELPD_, where smaller values indicate stronger support) and the SE of Δ_ELPD_ are shown for each cross‐validation exercise.

### Out‐of‐sample prediction and hotspot mapping for coho mortality

We generated coho mortality predictions for all Puget Sound subbasins where coho are known to occur, including the subbasins associated with the 51 spawning reaches that were surveyed for this study and an additional 1,481 unmonitored subbasins, whose mean area was 13.0 km^2^ (SE = 0.2). The vector‐based subbasin representation (WADOE 2011) was overlain with the same geospatial data layers used in the original analysis, and the covariates for each subbasin were calculated using the same methodology. Based on the model selection results, the “full” model with all landscape and precipitation effects was used. As in cross‐validation over sites, the posterior predictive distribution was conditioned on all available data; in particular, the landscape attributes from all subbasins (*S *=* *1,532) were allowed to inform the factor‐analytic component of the SEM. For these baseline predictions, summer and fall precipitation were set to their 2000–2011 averages (i.e., precipitation anomalies were set to zero).

## Results

**Figure 3:**
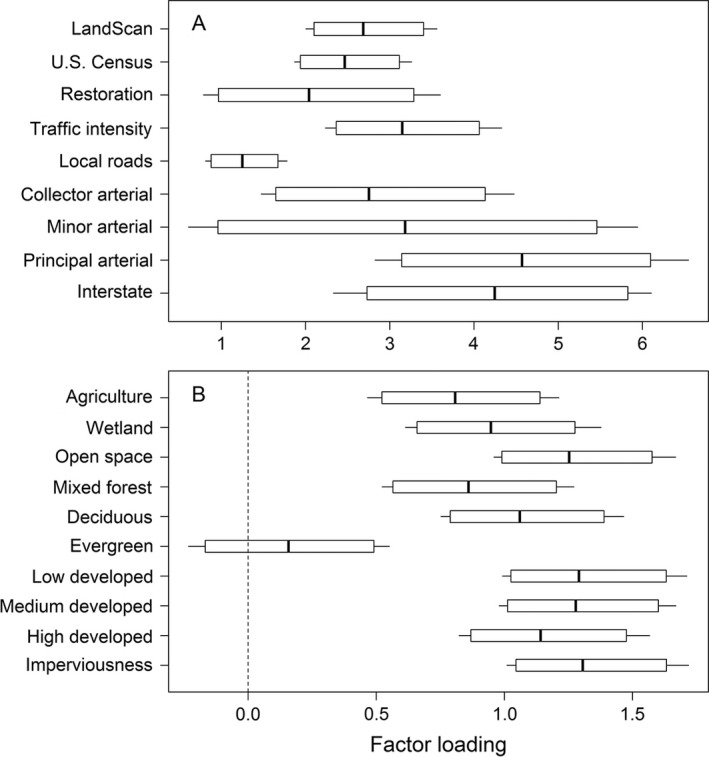
Posterior distributions of factor loadings relating land use/land cover variables to the latent variable (“urbanization”) in a structural equation model to predict coho pre‐spawn mortality. Variables were modeled as either (A) gamma‐distributed or (B) logistic normal, and loadings can be compared within but not between these classes. Because urbanization is positively associated with mortality risk, variables with positive loadings are also positively associated with mortality. Box plots show the posterior mean (thick line) and the 90% (box) and 95% (whiskers) credible intervals.

Structural equation modeling identified a single latent dimension of covariation among the 19 landscape indicators, representing an underlying “urbanization gradient.” Most landscape variables loaded positively on this latent factor in the full model. For example, human population density, traffic volume, density of highways and major arterial roads, and log ratios corresponding to percent cover of low, medium, and high development and impervious surfaces all had strongly positive loadings (Fig. 3). The lone exception was the log ratio for evergreen forest cover, whose loading had a posterior distribution that overlapped zero. We caution against comparing the relative magnitudes of loadings for gamma‐distributed (Fig. 3A) and logistic normal (Fig. 3B) variables because of the disparate transformations and likelihoods involved. Within each variable class, however, loadings can be interpreted as a relative measure of the responsiveness to urbanization. For example, arterial and interstate road density increase more strongly than local road density with overall urbanization, although the differences are modest compared to posterior uncertainty. This suggests that the relative density of vehicle traffic for different road classifications is more important than the cumulative landscape‐scale area of roads within a given classification. Consequently, despite a lower total area, more heavily used roads are more strongly associated with recurrent coho mortality.

**Figure 4:**
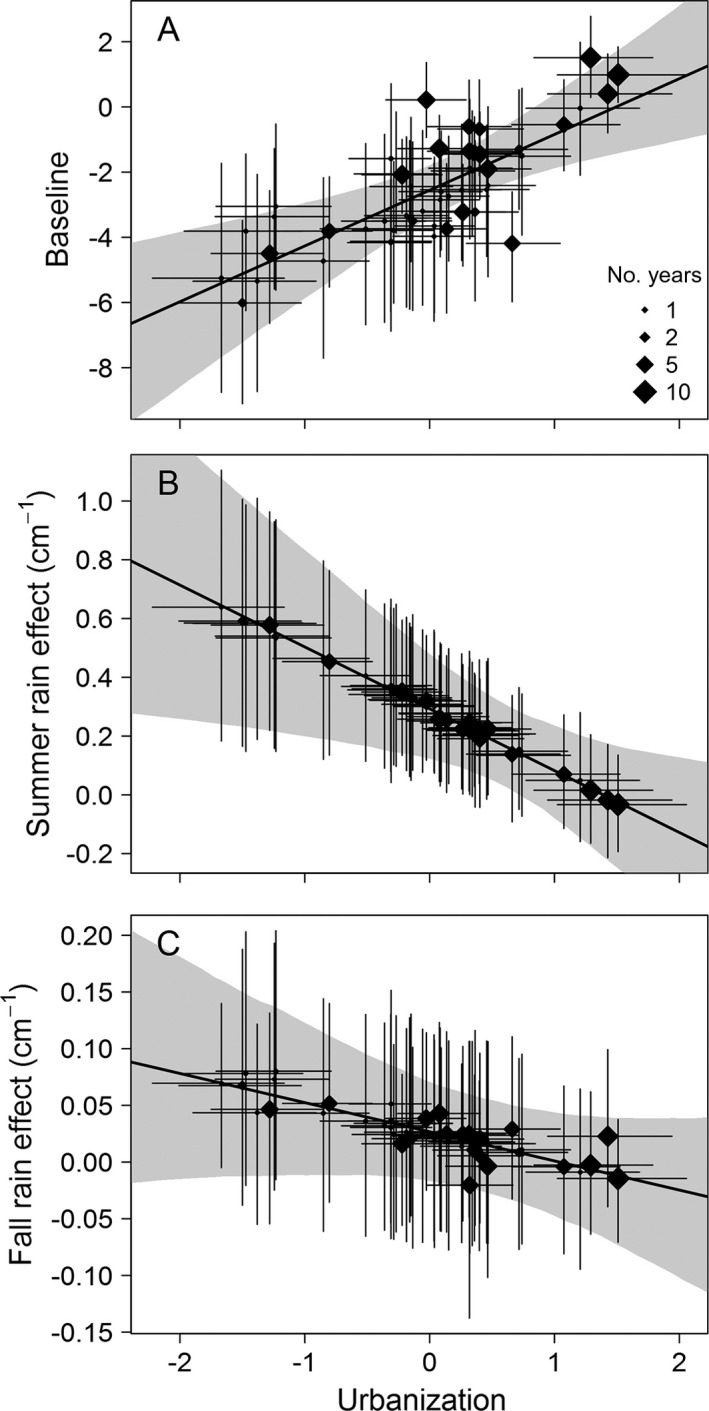
Models for the subbasin‐specific regression coefficients (the β^(*s*)^ in Eq. (4) and Fig. 2) in the GLMM that makes up one component of an overall SEM relating landscape and climate to coho pre‐spawn mortality risk. Each point is a coefficient in a logistic regression predicting annual mortality within a given subbasin. Point size corresponds to the number of years each subbasin was monitored. Each batch of coefficients is modeled as a function of the latent “urbanization” factor score in the corresponding subbasins, plus some residual error (subbasin‐specific random effects, the scatter of points around the regression line). Posterior uncertainty (95% credible intervals) in the coefficients and latent factor scores is represented by vertical and horizontal error bars, respectively, and uncertainty in the subbasin‐level regressions (95% credible intervals) is shown by gray envelopes. A positive effect of urbanization on the (A) intercept translates to a positive main effect of urbanization on mortality, while negative effects on the (B) summer and (C) fall precipitation slopes correspond to interactions whereby the per‐unit effect of rain on mortality risk declines with increasing urbanization.

The subbasin‐level model for the mortality regression coefficients revealed strong and interacting effects of landscape and climate on mortality risk. In the full model, the intercept of logit mortality probability ($\beta_{0}^{\left(s\right)}$) increased with urbanization, a main effect corresponding to overall higher rates of coho spawner mortality in more developed subbasins (Fig. 4A). Urbanization also modified the influence of seasonal rainfall. In the least developed (i.e., suburban and exurban) subbasins, summer precipitation was strongly associated with an increase in mortality risk ($\beta_{1}^{\left(s\right)}$ > 0). However, this association weakened along a gradient of urbanization, becoming indistinguishable from zero in the most developed subbasins (Fig. 4B). A similar pattern emerged for fall precipitation, although the evidence for an effect on mortality risk was equivocal; the 95% credible interval for $\beta_{2}^{\left(s\right)}$ overlapped zero even in the least developed subbasins. The influence of the urbanization gradient was also less evident, with more unexplained random‐effect variation around the regression line (Fig. 4C). As with summer rainfall, the marginal contribution of fall precipitation to coho mortality risk was negligible in the most urbanized subbasins, although overall mortality risk was highest in these subbasins.

**Figure 5:**
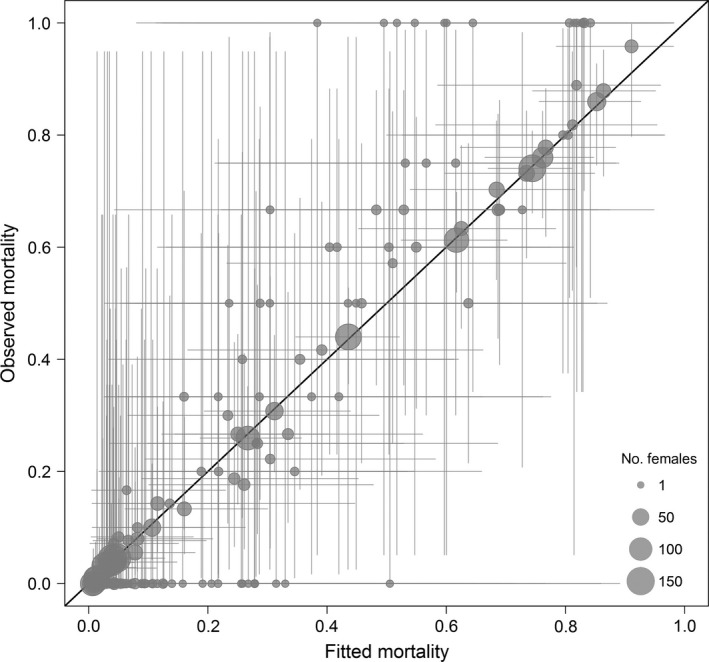
Fitted (posterior mean) and observed (sample proportion) probabilities of coho pre‐spawn mortality from a structural equation model incorporating the effects of landscape and climate. Each point is an annual observation of mortality, and the plot shows data from all subbasins. Point size indicates the number of female spawner carcasses sampled. Error bars show uncertainty in predictions (95% credible interval) and in sample estimates (95% confidence interval from the binomial distribution). The 1:1 line is shown for reference.

The data‐level regression in the GLMM‐like component of the SEM captured the primary patterns of variation in observed coho mortality across space and time (Fig. 5). Fitted (i.e., posterior mean) probabilities of spawner mortality from the full model generally tracked the observed mortality rates (*r *=* *0.91). The model tended to slightly underestimate mortality in the most urban subbasins where annual mortality rates are typically the highest (50–70%; Fig. 5). However, these discrepancies were small compared to uncertainty in both the predictions (posterior parameter uncertainty) and the field observations (binomial sampling error, which is greatest when small numbers of female carcasses were sampled).

Information criteria suggested considerable uncertainty in the ranking of the 18 candidate models. Differences among models in both WAIC and PSIS‐LOO were often dwarfed by their estimated standard errors (Table 1). In addition, Pareto smoothing diagnostics indicated that many of the PSIS‐LOO estimates were numerically unstable (Vehtari et al. 2015). These ambiguous results further motivated our use of direct simulation (i.e., cross‐validation) to estimate out‐of‐sample predictive performance. Although WAIC and PSIS‐LOO yielded different rankings, the top two models for each were the same (Table 1). We therefore focused on these, along with the full model and the null model (intercept only, no precipitation or urbanization effects). *K*‐fold cross‐validation over years showed the strongest support for a model with an effect of summer precipitation and a summer precipitation‐by‐urbanization interaction, but no main effect of urbanization and no effect of fall precipitation. The full model was ranked third and the null model was ranked last (Table 2). Again, however, differences in cross‐validation scores among models were often smaller than the associated standard errors, indicating substantial model selection uncertainty. *K*‐fold cross‐validation over monitored subbasins gave a much more decisive ranking, with the full model ranked first (Table 2). The second best model differed from the full model only in dropping the fall precipitation‐by‐urbanization interaction, and performed significantly worse (Δ = 8.31, SE = 2.68).

**Figure 6:**
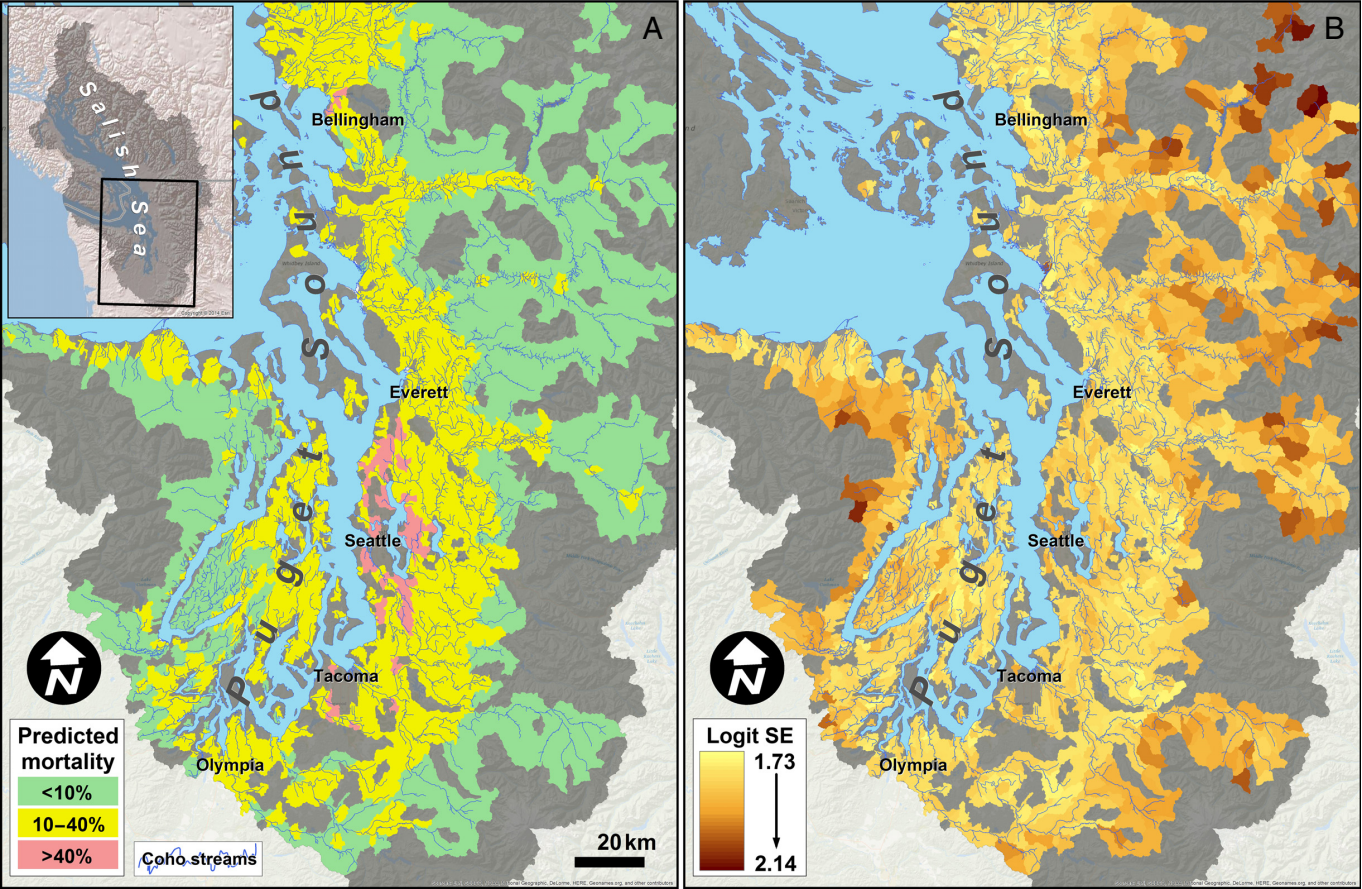
(A) Predicted mean spawner mortality using random draws from joint posterior distribution (incorporates parameter uncertainty). (B) Uncertainty (expressed as SE on the logit scale) calculated from posterior distribution for each estimate of mortality.

Baseline predictions of coho vulnerability for freshwater habitats across the entire Puget Sound basin, based on the full SEM, indicated high mortality risk concentrated around major urban population centers (Fig. 6A). Expected probabilities of mortality were >10% throughout much of the north‐south urban corridor surrounding Interstate 5, with pockets of risk as high as 54% in watersheds in the Seattle metropolitan area. More than half (58%) of the habitat currently available to coho in Puget Sound fell within the <10% predicted mortality category, 40% fell within the 10–40% category, and 2% fell within the >40% category (Fig. 6A). The precision of these predictions was relatively high in the urban corridor, as reflected in consistently low posterior standard deviations on the logit scale (Fig. 6B). Uncertainty increased in the least developed, higher‐elevation subbasins where few mortality monitoring data are available and mortality rates are predicted to be the lowest.

## Discussion

Migratory Pacific salmon return to spawn in the large river basins of western North America, where the current pace of urbanization is nearly twice that of the rest of the United States (Minnesota Population Center 2011). The negative ecological effects of development on salmon habitats have generally been viewed through a physical habitat lens, and the impacts of dams, water diversions, dredging, logging, diking, gravel mining, and many other human activities have been known for decades (NRC 1996, Ruckelshaus et al. 2002, Katz et al. 2007). Accordingly, federal and state investments in salmon habitat restoration have overwhelmingly addressed physical processes, e.g., restoring stream connectivity, increasing in‐stream flows, adding structure, etc. (Katz et al. 2007). Water quality improvements are a common objective but are usually limited to dissolved oxygen, temperature, and sediment (Barnas et al. 2015). We provide evidence here for a critical loss of spawners across much of the Puget Sound coho population segment, which is closely correlated with landscape‐scale measures of human population density and transportation infrastructure. Our findings are consistent with the hypothesis, supported by direct experimental evidence discussed below, that contaminants in stormwater runoff from the regional transportation grid likely cause these mortality events. Further, it will be difficult, if not impossible, to reverse historical coho declines without addressing the toxic pollution dimension of freshwater habitats.

A vexing challenge for many studies of landscape‐level impacts on ecological processes and species of concern is that landscape attributes covary at relevant spatial scales, making it difficult to disentangle their individual effects (Dormann et al. 2013). In a previous study of the urban coho mortality phenomenon (Feist et al. 2011), spatial multicollinearity across a small sample of subbasins produced high model selection uncertainty and unstable parameter estimates, even though candidate predictor variables were screened to avoid the most severe correlations. Moreover, this approach is an inefficient use of information, since the discarded variables might have helped to refine the predictions. In the present study, rather than pursue the difficult task of pinpointing causal pathways involving specific features of the built environment, we instead developed an integrative indicator of mortality risk, which we term the “urbanization gradient.” Bayesian structural equation modeling (SEM) is well suited to this task, providing a powerful and flexible framework that can extract the underlying signal (in the form of latent variables) from noisy multivariate data and model the directed relationships among these latent variables and observed outcomes (Arhonditsis et al. 2006, Grace et al. 2010, Lee and Song 2012). SEM, used as a form of supervised dimension reduction, offers an advantage over the two‐stage or unsupervised approach of regression on ordination axes because the latent factors derived from SEM are automatically weighted by their ability to predict the response variable. That is, the ordination is coupled to the prediction task—in our case, the hierarchical logistic regression model predicting coho mortality frequencies across space and time.

Predictive skill is an important criterion for models designed to support decision‐making, and the most appropriate tests of predictive ability depend on the context in which the model will be used. We found that different approaches to estimating out‐of‐sample predictive skill gave different answers about the relative performance of candidate models. These differences highlight the importance of considering spatial and temporal scale when building and evaluating complex hierarchical models. For example, information criteria such as WAIC and PSIS‐LOO are approximations to the posterior predictive density for each individual observation, if the model had been fitted to all the other observations (Vehtari and Ojanen 2012). These approximations are convenient because they can be computed directly from MCMC output without refitting the model, but they are sensitive to outliers and influential observations. In our case, information criteria generally favored models with a main effect of urbanization and a summer rainfall‐by‐urbanization interaction, but uncertainty in the estimates suggested that model rankings should be interpreted cautiously (Table 1). More to the point, actual applications of the model are more likely to entail predicting mortality risk in unmonitored basins or in multiple basins under future development or climate scenarios, rather than predicting isolated points in space and time. Cross‐validation (Hooten and Hobbs 2015) is a more direct, albeit computationally intensive way to simulate these predictive scenarios. Cross‐validation over years suggested that the main effect of urbanization did not clearly improve predictive skill (Table 2), which might seem surprising given the evident pattern of much higher mortality in urban streams than in less developed watersheds. However, when making predictions for “new” years within a given set of locations, the model can account for spatial variation with random effects informed by the available data from those same locations. By contrast, when the model was asked to predict mortality at “new” locations (cross‐validation over subbasins), its performance clearly suffered unless it explicitly accounted for the effects of urbanization (Table 2).

Our findings have practical applications for restoration practices in terms of avoiding ecological traps (Hale et al. 2015). The coho mortality phenomenon has been known since at least the late 1980s (Kendra and Willms 1990). However, it was studied more intensively after a restoration project (removal of migration barriers, e.g., culverts) in the 1990s unintentionally attracted coho to spawning areas where surface water quality was lethal (Scholz et al. 2011). Our landscape‐scale vulnerability projections can inform restoration planning operating at a local scale and avoid the similar creation of nuisance habitats (Dwernychuk and Boag 1972, Schlaepfer et al. 2002, Battin 2004, Robertson and Hutto 2006, Sih et al. 2011). The significance of ecological traps in salmon habitat restoration is becoming increasingly clear (Jeffres and Moyle 2012), and minimizing the potential for this in urban watersheds is particularly important given proportionally higher project costs.

Our current results are consistent with a growing body of evidence implicating motor vehicle‐derived contaminants as the cause of the coho spawner mortality phenomenon. The symptoms of coho die‐offs were recently reproduced under controlled conditions by exposing spawners to highway runoff, and this toxicity was removed when the same runoff was filtered through experimental soil columns to remove chemical pollutants (Spromberg et al. 2016). Although the present study was not designed to identify a specific “smoking gun” among the many correlated components of urban infrastructure, the factor loadings for different roadway types are consistent with these experimental results. Roads with the highest volume of traffic (highways and interstates) had higher factor loadings compared with less‐heavily traversed local roads, as would be expected if motor vehicles, rather than asphalt or pavement per se, are the source of the as‐yet‐unidentified chemical agent(s) linked to coho spawner mortality. However, our findings do not discount the well‐known negative impacts of imperviousness on urban stream health more generally, as extensively documented in Puget Sound (Booth et al. 2004, Alberti et al. 2007) and elsewhere throughout the world (Arnold and Gibbons 1996, Allan 2004, Schueler et al. 2009).

Field surveys initially indicated that rainfall may play a role in the year‐to‐year variation in spawner deaths (Scholz et al. 2011). The hypothesis was that prolonged periods of drought in the weeks or months prior to coho spawning season, followed by precipitation washing the concentrated contaminants into spawning streams, increased the magnitude of mortality. However, our present results surprisingly suggest that cumulative precipitation is only a factor in less urbanized basins and not related to mortality at all in the most urbanized areas. We propose that the most urbanized basins are relatively insensitive to periods of drought and subsequent rainfall because sufficient toxicants accumulate on roadways over relatively short periods of time, thereby minimizing the influence of antecedent dry intervals. In less developed basins, where contaminants are presumably deposited at lower rates, periods of drought in the summer followed by above‐average precipitation during the spawning season may influence spawner mortality rates. Consistent with this, a recent study exposed otherwise healthy adult coho to runoff from heavily used roadways such as highways and interstates, across multiple fall storm events (Spromberg et al. 2016). Despite antecedent dry intervals ranging from a few hours to more than a week, runoff from every storm caused 100% mortality. Our current modeling, together with this direct line of evidence, supports the conclusion that coho in more urbanized watersheds are vulnerable to non‐point source pollution irrespective of the timing, intensity, and frequency of storms. In the Pacific Northwest, global climate change is expected to reduce annual stream flows due to decreased winter snow pack and subsequent diminished summer snow melt (Luce et al. 2013). Lower in‐stream flows will likely result in less dilution of contaminants in receiving waters, thereby worsening pollution impacts on aquatic species and communities.

The innovation and implementation of clean water initiatives will invariably represent an important portfolio of conservation tools for coho salmon in Puget Sound and elsewhere in western North America (e.g., the greater Portland metropolitan area). In the United States, societal engagement and efforts to reduce urban non‐point source pollution have grown in recent years (Barbosa et al. 2012, Kaplowitz and Lupi 2012, Kim and Li 2016). There are myriad clean water strategies for this, both established and under development. In the built environment, these are collectively referred to as green stormwater infrastructure (GSI). The common goal is to slow, spread, and infiltrate stormwater, to reduce high flows (i.e., flooding) and filter pollutants (Sherrard et al. 2004, Elsaesser et al. 2011). In Puget Sound and elsewhere, initial bioinfiltration research has shown that simple and inexpensive soil columns can be very effective at removing chemical contaminants, thereby protecting the health of fish and aquatic invertebrates (McIntyre et al. 2014, 2015, 2016). As noted above, bioinfiltrating runoff from a high‐traffic urban arterial prevents lethal impacts on coho spawners (Spromberg et al. 2016). This suggests that GSI will be essential for (1) reducing the high rates of coho losses in subbasins that are currently urban or suburban and (2) preventing mortality in rural and forested subbasins where coho runs are presently healthy but vulnerable to future development pressures. While important, retrofitting the built environment is expensive, and it will be easier to incorporate clean water strategies into future growth (Hughes et al. 2014). Natural resource decision making will be based, in part, on spatially explicit tools (Shamsi et al. 2014), and this can be guided by the coho die‐off hotspot vulnerability maps that we have generated here.

Our methods are transferrable, since they are based on geospatial data that are available nationally or globally, and can be applied to other management contexts where urban sprawl threatens species conservation (McQueen et al. 2010, Marmonier et al. 2013). This includes, for example, assessments of coho vulnerability in other metropolitan areas of northwestern North America, now and in the future. Alternative futures analyses, a cornerstone of urban planning, weigh options related to the transportation grid as well as residential, industrial, and commercial development. In the Puget Sound region, this includes growth management for human population density and increasing imperviousness in the coming decades (e.g., Bolte and Vache 2010). Our predictive models could inform alternative growth management scenarios, to identify where non‐point source pollution control measures will be most needed to ensure long‐term coho conservation and, by extension, the integrity and resiliency of freshwater and nearshore marine communities.

## Conclusions

Extensive fish kills have become rare in the United States in the decades since the Clean Water Act was passed to control point‐source pollution. The most important water quality threat to aquatic systems now is non‐point source pollution (Scholz and McIntyre 2015). The coho mortality phenomenon is one of the few contemporary examples of urban stormwater causing the overt death of a widely distributed keystone species with high societal value, both economically and culturally. By sharpening our ability to predict where coho are dying, the present study brings us a step closer to understanding the underlying cause, and highlights the fact that 40% of the total area of Puget Sound river basins that support coho are predicted to have adult mortality rates that substantively increase the risk of local population extinction. Finally, we have shown where green stormwater infrastructure and other clean water strategies are most needed at the landscape and basin scales. In the coming years, coho salmon spawners will be a sentinel for the success, or failure, of these efforts.
