# Supplementary material for: Roads to ruin: conservation threats to a sentinel species across an urban gradient
Source: Ecol Appl. 2017 Oct 18;27(8):2382–96. doi: 10.1002/eap.1615 (PMC6084292; doi:10.1002/eap.1615)
Supplement: Supplementary file 1 [file EAP-27-2382-s001.PDF]

Feist et al. Roads to ruin: conservation threats to a sentinel species across an urban gradient. ***Ecological Applications***

Appendix S1

Additional details of data analysis methods.

Author(s)

Blake E. Feist[1*], Eric R. Buhle[2], David H. Baldwin[3], Julann A. Spromberg[3], Steven E. Damm[4],
Jay W. Davis[4], and Nathaniel L. Scholz[3]

[1] Conservation Biology Division
Northwest Fisheries Science Center
National Marine Fisheries Service
NOAA
2725 Montlake Blvd E, Seattle, WA 98112
USA.

[2] Quantitative Consultants, Inc.
Under contract to Northwest Fisheries Science Center
National Marine Fisheries Service
NOAA
2725 Montlake Blvd E, Seattle, WA 98112
USA.

[3] Environmental and Fisheries Sciences Division
Northwest Fisheries Science Center
National Marine Fisheries Service
NOAA
2725 Montlake Blvd E, Seattle, WA 98112
USA.

[4] Washington Fish and Wildlife Office
United States Fish and Wildlife Service
510 Desmond Dr. SE
Lacey, WA 98392
USA

Section A1: Geospatial data layer processing and overlays with study basins

Geospatial data layers are referred to by the covariate names listed in Table 2 and are underlined in the text.

We calculated a density for the gridded human population data (LandScan) by summing the number of people for each subbasin, divided by subbasin area (people/km^2 of stream subbasin). Irregularly shaped human population polygons (U.S. Census 2000 and 2010) were aggregated at the block scale (finest available). We corrected for irregular polygons that intersected subbasin boundaries by calculating the proportional polygon area within a given subbasin and multiplied this by the population value. Finally, we averaged the 2000 and 2010 census data for each subbasin. Restoration locations were reported as points and were summed within each subbasin and divided by subbasin area (restoration projects/km^2). Traffic intensity data were reported as annual average daily traffic (AADT) by roadway line features, limited to arterials, collectors (including ramps), highways and interstates. We converted this to area weighted mean (AWM) traffic intensity by calculating length-weighted mean traffic intensity and dividing this by subbasin area. We calculated the density of each of the five roadway categories (m/km^2 of stream subbasin) and the proportion of each of the nine land use and land cover categories within a given subbasin. Mean values were then determined across the three years of available data (2001, 2006 and 2010). For the percent imperviousness gridded data layer, we calculated an AWM value for each subbasin using the following equation:

$$\overline{x} = \frac{\sum_{i=1}^{n} w_i x_i}{\sum_{i=1}^{n} w_i}, \qquad (A1)$$

where x is the imperviousness value for a given group of grid-cells, and w is the number of grid cells that fall within a given site subbasin. Given the fine spatial grain (30 m grid cell size), we did not correct for grid cells that fell on subbasin boundaries. Finally, we averaged the 2001, 2006 and 2010 data for each subbasin.

Precipitation was grouped monthly for the summer (July and August) and fall (October and November) seasons, and then summarized annually by subbasin from 2000 to 2011. We calculated an AWM precipitation value for each subbasin using the following equation:

$$\overline{x} = \frac{\sum_{i=1}^{n} a_i x_i}{\sum_{i=1}^{n} a_i} , \qquad \text{(A2)}$$

where x is the precipitation value for a given group of grid-cells, and a is the total area (m^2) of grid cells that fall within a given site subbasin. The precipitation data were collected over a set of larger grids (~3.1 x 4.6 km), so we corrected for grid cell boundary intersection by calculating the proportion of each grid cell's area that fell within a given subbasin, and multiplied the proportion by the precipitation value.

Section A2: Parameter estimation

Before specifying the full posterior distribution we must consider the well-known problem of identifiability in the factor-analytic component of the SEM and its implications for Bayesian inference using MCMC. It will be convenient to work with the more compact matrix form of the model rather than the elementwise notation used in Eqs.

1 and 2 in the main text. The factor model for normally distributed observations (Eq. 1) can be written as

$$\begin{aligned} &\mathbf{X} = \mathbf{A_0} + \mathbf{Z}\mathbf{A}^{\mathsf{T}} + \boldsymbol{\varepsilon} \\ &\mathbf{Z}_{s,1:L} \sim \mathrm{MVN}(\mathbf{0},\mathbf{I}) \ , \\ &\boldsymbol{\varepsilon}_{s,1:D} \sim \mathrm{MVN}(\mathbf{0},\boldsymbol{\Sigma}) \end{aligned} \tag{A3}$$

where $\mathbf{X}$ is the $S\times D$ matrix of landscape data, $\mathbf{A_0}$ is an $S\times D$ matrix of intercepts formed by stacking S copies of the row vector $[a_{01}, \ldots, a_{0D}]$, $\mathbf{Z}$ is the $S\times L$ matrix of latent factors with corresponding $D\times L$ loading matrix $\mathbf{A}$, $\mathbf{I}$ is the identity matrix, and the $S\times D$ residual error matrix $\boldsymbol{\varepsilon}$ has mutually independent rows each following a multivariate normal distribution with covariance matrix $\boldsymbol{\Sigma} = \mathrm{diag}(\boldsymbol{\sigma}) = \mathrm{diag}(\sigma_1, \ldots, \sigma_D)$. The model for non-Gaussian observations (Eq. 2) is analogous, but the linear regression is on the link scale and the multivariate normal is replaced with the appropriate exponential family distribution f:

$$\begin{aligned} &g(\boldsymbol{\mu}) = \mathbf{A_0} + \mathbf{Z}\mathbf{A}^{\mathsf{T}} \\ &\mathbf{Z}_{s,1:L} \sim \mathrm{MVN}(\mathbf{0},\mathbf{I}), \\ &\mathbf{X} \sim f(\boldsymbol{\mu},\boldsymbol{\Phi}) \end{aligned} \tag{A4}$$

where the factor and loading notation follows Eq. A3 and $f(\boldsymbol{\mu},\boldsymbol{\Phi})$ denotes a vectorized pdf with $S\times D$ mean matrix $\boldsymbol{\mu}$ and dispersion matrix $\boldsymbol{\Phi}$. Our analysis includes both Gaussian and non-Gaussian landscape variables, but to avoid cluttering the notation we do not explicitly distinguish between the two respective sets of loadings and intercepts.

For the factor values and loadings to be identifiable, the dimension of the factor space must be less than half the dimension of the observed landscape variables, $L < D/2$ (Geweke and Zhou 1996). Even if this condition is met, there is still a rotational indeterminacy because the factor matrix **Z** can be multiplied by any orthogonal matrix, and the loading matrix **A** by its inverse, without changing the linear predictor and thus the likelihood of the data. Geweke and Zhou (1996) make the model identifiable by constraining the loading matrix to be lower triangular with positive diagonal elements. This parameterization, which is widely used in Bayesian factor analysis, eliminates the trivial multimodality in the likelihood (i.e., different rotations of parameter space with identical likelihoods), but constraining the diagonal elements to be positive *a priori* can induce nontrivial multimodality (i.e., modes that are not identical up to an orthogonal rotation) (Erosheva and Curtis 2011). To avoid this, we constrained **A** to be lower triangular but did not restrict the sign of the diagonal elements *a priori*. This induces a reflectional invariance because each factor and its corresponding loadings can be multiplied by -1 without changing the likelihood. We resolved this indeterminacy by post-processing the MCMC output. For each draw from the posterior, we multiplied the factor scores $\mathbf{Z}_{1:S,l}$ and loadings $\mathbf{A}_{1:D,l}$ for each factor l by the sign of the corresponding diagonal element a_{ll}, so the resulting matrix of loadings satisfies Geweke and Zhou's (1996) positive-diagonal constraint as well as the lower-triangular form. As a result, the MCMC sampler "sees" a trivially multimodal posterior distribution (with modes that are identical up to a reflection), which could potentially lead to inefficient sampling; however, we did not find that this caused any problems in practice with the Hamiltonian Monte Carlo algorithm.

The joint posterior distribution is given by

$$
\begin{aligned}
&P\left(\mathbf{A_0},\mathbf{A},\mathbf{Z},\boldsymbol{\sigma},\boldsymbol{\Phi},\boldsymbol{\Gamma},\boldsymbol{\sigma_\beta},\boldsymbol{\beta},\sigma_\delta,\mathbf{p}\mid\mathbf{X},\mathbf{y},\mathbf{n},\mathrm{ppt}_{\mathrm{su}},\mathrm{ppt}_{\mathrm{fa}}\right)\propto\\
&\quad P\left(\mathbf{A_0},\mathbf{A},\boldsymbol{\sigma},\boldsymbol{\Phi},\boldsymbol{\Gamma},\boldsymbol{\sigma_\beta},\sigma_\delta\right)\\
&\quad\times\mathrm{N}\left(\mathbf{Z}\mid\mathbf{0},\mathbf{I}\right)\\
&\quad\times\mathrm{N}\left(\mathbf{X}_{\mathrm{N}}\mid\mathbf{Z},\mathbf{A_0},\mathbf{A},\boldsymbol{\sigma}\right)\\
&\quad\times\mathrm{G}\left(\mathbf{X}_{\mathrm{G}}\mid\mathbf{Z},\mathbf{A_0},\mathbf{A},\boldsymbol{\Phi}\right)\\
&\quad\times\mathrm{N}\left(\boldsymbol{\beta}\mid\mathbf{Z},\boldsymbol{\Gamma},\boldsymbol{\sigma_\beta}\right)\\
&\quad\times\mathrm{N}\left(\mathrm{logit}\left(\mathbf{p}\right)\mid\boldsymbol{\beta},\mathrm{ppt}_{\mathrm{su}},\mathrm{ppt}_{\mathrm{fa}},\sigma_\delta\right)\\
&\quad\times\mathrm{Bin}\left(\mathbf{y}\mid\mathbf{n},\mathbf{p}\right)
\end{aligned}
\tag{A5}
$$

Here $\mathbf{X}_{\mathrm{N}}$ and $\mathbf{X}_{\mathrm{G}}$ are the submatrices of **X** that contain normal and gamma-distributed landscape variables, and N(·|·) and G(·|·) denote the normal and gamma likelihoods, respectively (Eqs. 1, 2 in the main text). The matrix **Γ** contains the among-subbasin regression coefficients γ_{kl}, which together with the random-effect standard deviations $\boldsymbol{\sigma_\beta}$ determine the distribution of the subbasin-specific logistic regression coefficients **β** (Eq. 4 in the main text). The last two factors are the likelihood of the data-level random effects (overdispersion terms) on the logit of mortality risk **p** and the likelihood of the observed mortality frequencies **y** given the carcass sample sizes **n** (Eq. 3 in the main text). We used vague prior distributions for the hyperparameters (the second line of Eq. A5). All standard deviations were given Unif(0,10) priors and all factor loadings (elements of **A**), factor intercepts (elements of $\mathbf{A}_0$) and subbasin-level intercept and slope hyperparameters (elements of **Γ**) were given N(0,10) priors.

Section A3: Model selection

For cross-validation over years, we excluded all spawner survey data from each year (2000-2011) in succession and then fit the candidate model to the remaining data, calculating the log posterior predictive density for each of the excluded observations (Vehtari et al. 2015). For cross-validation over sites, to reduce the computational burden we randomly partitioned the 51 subbasins into 10 strata containing roughly equal sample sizes of spawner data. We excluded each of the strata in turn and calculated the log predictive density for the excluded observations conditional on the fit to the data from the remaining sites. This procedure assumes landscape and precipitation data are available for all subbasins regardless of whether they have been monitored for coho mortality. In both exercises, the cross-validation score was the log predictive density summed across all mortality observations (see Computer Code and Data supplement, Vehtari et al. 2015).

Table S1. Data sources and years when data were collected for each of the 51 site subbasins. First value in cell is the total number of confirmed female prespawn mortalities for any given year and the second corresponding value is the total number of female spawners for that year.

Site Name	2000	2001	2002	2003	2004	2005	2006	2007	2008	2009	2010	2011
Barker[1]					5/12				0/1			
Big Scandia[1]					1/1		1/5		1/2	1/3		
Blackjack[1]					55/125	5/50	0/1	1/1	0/2	0/1	0/3	
Bosworth[2]				0/7								
Canyon[2]				2/25								
Catherine[2]				1/36								
Cherry[3]						4/10						
Chico[1]					23/24	4/15	2/2	2/2	2/3		3/5	
Church[4]												1/5
Clear WF[1]					1/4							
Cool[1]						0/94	1/14		0/35	0/11	0/2	
Curley[1]					27/104	8/30	0/2	6/9	0/2			
Curley Trib[1]					1/1	6/9	0/6	1/1	0/3	0/12		
Des Moines[5]					19/30							
Dickerson[1]						0/1	0/2	1/2	2/36	1/12		
Dogfish[1]					4/30	3/10	0/1	1/2	0/1	1/3		
Dogfish NF[1]					0/19	1/6	0/9	1/13	0/3	0/1		
Dry[2]				2/70								
Dubuque[2]				0/7								
Eager Beaver[2]				5/132								
EF Griffin[2]				4/28								
Fauntleroy[5]	3/12	2/9	0/1			3/4		3/4				
Fish[4]												7/153
Fortson[5]			1/114									
Gorst[1]					3/16	0/8	0/1	1/1	4/7	3/5	2/4	
Gorst Trib[1]					0/5					0/2		
Grizzly[2]				7/161								
Happy Hollow[4]												0/7

Site Name	2000	2001	2002	2003	2004	2005	2006	2007	2008	2009	2010	2011
Harris[2]				0/5								
Harris B[2]				0/4								
Harris C[2]				1/10								
Harris D[2]				2/5								
Index[2]				4/125								
Jarstad[1]							0/1	0/10				
Johnson[1]					1/1	0/1	3/5	1/3		0/1		
Lake[2]				2/44								
Lewis[2]				1/7								
Longfellow[5]	100/135	68/111	49/57	12/18	8/9	57/75	4/4	30/41	8/12	28/36		
Lost[1]					0/1	0/1	0/15	2/3	0/5	0/2		
MF Quilceda[2]				0/109								
Parish[1]								0/9	0/1	0/2	0/1	
People's[2]				1/114								
Pipers[5]	3/17	26/37	6/10	0/1	1/3	3/4	9/9	1/5				
Pond[2]				0/58								
Ross[2]				0/5								
Son of Deer[2]				0/5								
Thornton[5]	29/33	9/11	4/5	2/2	1/1	4/8	4/4	4/5	2/2			
Valhalla[4]												0/6
Weiss[2]				0/48								
Wildcat[1]					1/2	0/3	1/26	4/8	16/52	0/4	2/6	
Wildcat Trib[1]							0/4			0/4		

[1]Suquamish Tribe, Port Madison Indian Reservation, WA (unpublished data courtesy of Jon Oleyar)
[2]Wild Fish Conservancy (Washington Trout 2004)
[3]Wild Fish Conservancy (Wild Fish Conservancy 2008)
[4]Stillaguamish Tribe, Arlington, WA (unpublished data courtesy of Jody Brown)
[5]NOAA Fisheries (Scholz et al. 2011)

Table S2. Descriptions, types and citations for geospatial data layers used in spatial analyses. Each covariate was calculated for each of the 51 site basins.

Covariate	Description	Units	Citation
LandScan	Human population density based on LandScan gridded data in 2011	people/km^2	Bright et al. (2012)
U.S. Census 2000	Human population density based on US Census block data in 2000	people/km^2	U.S. Census Bureau (2001)
U.S. Census 2010	Human population density based on US Census block data in 2010	people/km^2	U.S. Census Bureau (2010)
Restoration	Density of all restoration site types for all years	sites/km^2	NMFS (2014)
Traffic intensity	Annual mean daily traffic	cars/km/km^2	WSDOT (2012)
Local roadways	Density of functional class 5 roads (all other roads)	m of road/km^2	HSIP (2013)
Collector arterial roadways	Density of functional class 4 roads (roads that provide for a high volume of traffic movement at moderate speeds between neighborhoods)	m of road/km^2	HSIP (2013)
Minor arterial roadways	Density of functional class 3 roads (roads that interconnect Level 2 roads and provide a high volume of traffic movement at a lower level of mobility than Level 2 roads)	m of road/km^2	HSIP (2013)
Principle arterial roadways	Density of functional class 2 roads (roads with very few, if any speed changes, and provide high volume, high speed traffic movement. Typically used to channel traffic to and from Level 1 roads)	m of road/km^2	HSIP (2013)
Interstate roadways	Density of functional class 1 roads (roads with very few, if any speed changes, typically controlled access, and provide high volume, maximum speed movement between and through major metropolitan areas)	m of road/km^2	HSIP (2013)
Agriculture land use	Proportion of site subbasin comprised of agricultural category in 2001, 2006 and 2010. Includes cultivated crops (6), and pasture/hay (7)	Proportion	NOAA (2013)
Wetland land cover	Proportion of site subbasin comprised of wetland category in 2001, 2006 and 2010. Includes palustrine forested- (13), palustrine scrub/shrub- (14), and	Proportion	NOAA (2013)

Covariate	Description	Units	Citation
	palustrine emergent-wetland subcategories (15)		
Open space land use	Proportion of site subbasin comprised of developed open space category in 2001, 2006 and 2010 (5)	Proportion	NOAA (2013)
Mixed forest land cover	Proportion of site subbasin comprised of mixed forest category in 2001, 2006 and 2010 (11)	Proportion	NOAA (2013)
Evergreen forest land cover	Proportion of site subbasin comprised of evergreen forest category in 2001, 2006 and 2010 (10)	Proportion	NOAA (2013)
Deciduous forest land cover	Proportion of site subbasin comprised of deciduous forest category in 2001, 2006 and 2010 (9)	Proportion	NOAA (2013)
Low intensity developed land use	Proportion of site subbasin comprised of low intensity developed category in 2001, 2006 and 2010 (4)	Proportion	NOAA (2013)
Medium intensity developed land use	Proportion of site subbasin comprised of medium intensity developed category in 2001, 2006 and 2010 (3)	Proportion	NOAA (2013)
High intensity developed land use	Proportion of site subbasin comprised of high intensity developed category in 2001, 2006 and 2010 (2)	Proportion	NOAA (2013)
Imperviousness	AWM percent developed imperviousness in 2001, 2006 and 2011	Percent	Xian et al. (2011), USGS (2014)
Summer precipitation	AWM cumulative rainfall from Jul – Aug, by year from 2000 to 2011	mm/km^2	Daly et al. (2012)
Fall precipitation	AWM cumulative rainfall from Oct - Nov, by year from 2000 to 2011	mm/km^2	Daly et al. (2012)

Supplementary Literature Cited

Bright, E. A., P. R. Coleman, A. N. Rose, and M. L. Urban. 2012. LandScan 2011. Oak Ridge National Laboratory, Oak Ridge, TN.

Daly, C., W. Gibson, M. Doggett, J. Smith, and G. Taylor. 2012. Near-real-time monthly high-resolution precipitation climate data set for the conterminous United States. The PRISM Climate Group, Oregon State University, Corvallis, OR.

Erosheva, E. A., and S. M. Curtis. 2011. Dealing with Rotational Invariance in Bayesian Confirmatory Factor Analysis. Tech Report 589, Department of Statistics, University of Washington, Seattle, WA.

Geweke, J., and G. Zhou. 1996. Measuring the Pricing Error of the Arbitrage Pricing Theory. Review of Financial Studies **9**:557-587.

HSIP. 2013. Homeland Security Infrastructure Program Gold 2013. National Geospatial - Intelligence Agency. Distributed by Ventyx, Boulder, CO.

NMFS. 2014. Pacific Northwest salmon habitat project database. National Marine Fisheries Service, Northwest Fisheries Science Center, Seattle, WA.

NOAA. 2013. NOAA's Coastal Change Analysis Program (C-CAP) 1992 to 2010 Regional Land Cover Change Data - Coastal United States. National Ocean Service (NOS), Office for Coastal Management (OCM), Charleston, SC.

Scholz, N. L., M. S. Myers, S. G. McCarthy, J. S. Labenia, J. K. McIntyre, G. M. Ylitalo, L. D. Rhodes, C. A. Laetz, C. M. Stehr, B. L. French, B. McMillan, D. Wilson, L. Reed, K. D. Lynch, S. Damm, J. W. Davis, and T. K. Collier. 2011. Recurrent die-offs of adult coho salmon returning to spawn in Puget Sound lowland urban streams. PLoS ONE **6**:e28013.

U.S. Census Bureau. 2001. 2000 Census Block, 2010 TIGER/Line Shapefile. U.S. Department of Commerce, U.S. Census Bureau, Geography Division, Geographic Products Branch, Washington, DC.

U.S. Census Bureau. 2010. 2010 Census Block, 2010 TIGER/Line Shapefile. U.S. Department of Commerce, U.S. Census Bureau, Geography Division, Geographic Products Branch, Washington, DC.

USGS. 2014. NLCD 2011 Percent Developed Imperviousness (2011 Edition). U.S. Geological Survey, Sioux Falls, SD.

Vehtari, A., A. Gelman, and J. Gabry. 2015. Practical Bayesian Model Evaluation Using Leave-One-out Cross-Validation and WAIC.

Washington Trout. 2004. Land use and coho prespawning mortality in the Snohomish watershed, Washington. EPA agreement ID MM97061201-0.

Wild Fish Conservancy. 2008. Coho prespawning mortality assessment in Washington and Oregon. EPA Assistance Agreement ID: X5-96007101-0.

WSDOT. 2012. Highway Performance Monitoring System. Washington State Department of Transportation, Olympia, WA.

Xian, G., C. Homer, J. Demitz, J. Fry, N. Hossain, and J. Wickham. 2011. Change of impervious surface area between 2001 and 2006 in the conterminous United States. Photogrammetric Engineering and Remote Sensing **77**:758-762.
