# Supplementary material for: Roads to ruin: conservation threats to a sentinel species across an urban gradient
Source: Ecol Appl. 2017 Oct 18;27(8):2382–96. doi: 10.1002/eap.1615 (PMC6084292; doi:10.1002/eap.1615)
Supplement: Supplementary file 3 [file EAP-27-2382-s003.pdf]

Feist et al. Roads to ruin: conservation threats to a sentinel species across an urban gradient. *Ecological Applications*

Data S1

Code and data files for structural equation modeling.

Author(s)

Blake E. Feist[1*], Eric R. Buhle[2], David H. Baldwin[3], Julann A. Spromberg[3], Steven E. Damm[4],
Jay W. Davis[4], and Nathaniel L. Scholz[3]

[1] Conservation Biology Division
Northwest Fisheries Science Center
National Marine Fisheries Service
NOAA
2725 Montlake Blvd E, Seattle, WA 98112
USA.

[2] Quantitative Consultants, Inc.
Under contract to Northwest Fisheries Science Center
National Marine Fisheries Service
NOAA
2725 Montlake Blvd E, Seattle, WA 98112
USA.

[3] Environmental and Fisheries Science Division
Northwest Fisheries Science Center
National Marine Fisheries Service
NOAA
2725 Montlake Blvd E, Seattle, WA 98112
USA.

[4] Washington Fish and Wildlife Office
United States Fish and Wildlife Service
510 Desmond Dr. SE
Lacey, WA 98392
USA

File list (files found within DataS1.zip)

```
lulc.roads.labels.csv
spatial.data.csv
spatial.data.predict.csv
spawner.data.csv
cohoPSManalyses.R
cohoPSM_SEM_Stan.stan
```

Description

`lulc.roads.labels.csv` – Descriptive variable names for boxplot labels.

`spatial.data.csv` – Subbasin (`site`) and watershed names for the 51 subbasins included in the retrospective analysis of pre-spawning mortality, with associated climatic variables (annual summer and fall precipitation [mm]) and land-use variables (see Table A2 for variable definitions and units).

`spatial.data.predict.csv` – See `spatial.data.csv`; this file includes 2977 subbasins where coho mortality was not measured, but where mortality risk predictions are desired.

`spawner.data.csv` – Coho pre-spawning mortality observations by subbasin (`site`) and year. The number of female carcasses examined is `n` and the number of mortalities is `n.psm`.

`cohoPSManalyses.R` – R script containing all code used in the analyses described in the paper, including data manipulation, model fitting and model selection, cross-validation, and plotting.

`cohoPSM_SEM_Stan.stan` – Stan code specifying the SEM, which can be called from R using the `rstan` library and commands given in `cohoPSManalyses.R`.
